# SIDD: A Semantically Integrated Database towards a Global View of Human Disease

**DOI:** 10.1371/journal.pone.0075504

**Published:** 2013-10-11

**Authors:** Liang Cheng, Guohua Wang, Jie Li, Tianjiao Zhang, Peigang Xu, Yadong Wang

**Affiliations:** Center for Bioinformatics, School of Computer Science and Technology, Harbin Institute of Technology, Harbin, Heilongjiang, China; The Centre for Research and Technology, Hellas, Greece

## Abstract

**Background:**

A number of databases have been developed to collect disease-related molecular, phenotypic and environmental features (DR-MPEs), such as genes, non-coding RNAs, genetic variations, drugs, phenotypes and environmental factors. However, each of current databases focused on only one or two DR-MPEs. There is an urgent demand to develop an integrated database, which can establish semantic associations among disease-related databases and link them to provide a global view of human disease at the biological level. This database, once developed, will facilitate researchers to query various DR-MPEs through disease, and investigate disease mechanisms from different types of data.

**Methodology:**

To establish an integrated disease-associated database, disease vocabularies used in different databases are mapped to Disease Ontology (DO) through semantic match. 4,284 and 4,186 disease terms from Medical Subject Headings (MeSH) and Online Mendelian Inheritance in Man (OMIM) respectively are mapped to DO. Then, the relationships between DR-MPEs and diseases are extracted and merged from different source databases for reducing the data redundancy.

**Conclusions:**

A semantically integrated disease-associated database (SIDD) is developed, which integrates 18 disease-associated databases, for researchers to browse multiple types of DR-MPEs in a view. A web interface allows easy navigation for querying information through browsing a disease ontology tree or searching a disease term. Furthermore, a network visualization tool using Cytoscape Web plugin has been implemented in SIDD. It enhances the SIDD usage when viewing the relationships between diseases and DR-MPEs. The current version of SIDD (Jul 2013) documents 4,465,131 entries relating to 139,365 DR-MPEs, and to 3,824 human diseases. The database can be freely accessed from: http://mlg.hit.edu.cn/SIDD.

## Introduction

Biological database integration has been a significant research domain, because of its intrinsic challenges in data standardization, ontology development and knowledge management. Many gene-centric [1], [2] and protein-centric [3]–[5] databases have been successfully developed and integrated. However, the development of disease-centric database has yet up to the desired standard.

Over the last decade, many disease-related molecular, phenotypic and environmental features (DR-MPEs) in terms of the human genes, non-coding RNAs, genetic variations, drugs, environments, etc., have been studied and disease-associated databases have been developed to understand the nature of disease. For example, Online Mendelian Inheritance in Man (OMIM) [6] is the main repository of genetic information for medelian disorders. Human Metabolome Database (HMDB) [7] contains metabolite associated disease currently. miR2Disease [8] and human microRNA disease database (HMDD) [9] have been developed to explore the relationships between microRNA and disease. The NHGRI GWAS Catalog (http://www.genome.gov/gwastudies) collects all GWAS data for examining the relationships between SNPs and diseases. Human Phenotype Ontology (HPO) [10] has been developed for representing individual phenotypic anomalies and has annotated all clinical entries in OMIM. Dr.VIS [11] maintains human disease-related virus data. BRENDA [12] provides information about diseases connected to anomalous enzyme function. dbCRID [13] is a comprehensive database of human chromosomal rearrangement events and their associated diseases. These databases in fact only focus on one or two types of DR-MPEs; it is important to integrate multiple types of DR-MPEs for a more comprehensive understanding of disease. The organization of disease terminologies in some databases is based upon an open source medical vocabulary, such as Disease Ontology (DO) [14], Medical Subject Headings (MeSH) [15], OMIM. In other databases the disease vocabularies are built by themselves. The differences in vocabularies among these databases are the primary challenge in understanding multiple types of DR-MPEs.

In order to associate genetic and genomic data with human disease, a robust disease ontology is required [16], [17]. To standardize human disease annotations in biomedical databases, DO has been established for the classification of disease from the clinical perspective of etiology and its specific tissue/organ location. The DO is organized into eight main diseases categories anchored by traceable, stable identifiers (DOIDs) [14]. DO semantically integrates multiple disease and medical vocabularies (MeSH, ICD (International Classification of Diseases), OMIM and SNOMED CT (Systematized Nomenclature of Medicine Clinical Terms)) and allows the cross reference with these vocabularies [14]. Davis et al. [18] have merged MeSH and OMIM to MEDIC (MErged DIsease voCabulary), which fuses the hierarchical structure of disease in MeSH and the detailed disease description in OMIM. This vocabulary has been used to annotate and infer chemical-disease and gene-disease relationships in the Comparative Toxicogenomics Database (CTD) [19]. Névéol et al. [17] proposed a method to link the multiple disease-related resources through UMLS [20], and map 467 Gene Reviews with 1,581 disease records. In spite of some advances in integrating disease-related resources, they don’t provide a user interface to browse the database. Recently, Xu et al. [21], [22] have proposed an automatic method — Disease Ontology Annotation Framework to provide a comprehensive annotation of the human genome, and have designed a Disease and Gene Annotations (DGA) database for comprehensive and integrative annotation of the human genes in disease network. Though disease-gene association and their association network have been established in DGA, it didn’t provide a global disease view for browsing multiple types of disease-related factors because the disease vocabularies in different databases haven’t been integrated.

In this study, a semantically integrated database (SIDD) is presented. It provides a web interface for understanding disease from many biological levels. 18 disease-related databases associated with multiple types of DR-MPEs have been integrated in SIDD. SIDD's disease terminologies are based upon DO, since this is the first vocabulary that organizes terms around the concept of disease, and semantically integrates multiple existing ontologies [14]. To unify different disease vocabularies of these databases, a mapping strategy for establishing association from DO to them has been investigated. Using SIDD, the associations among DR-MPEs that affect the same disease in different databases can be collectively investigated. Furthermore, diseases and their co-related DR-MPEs have been visualized in network. The current version of SIDD, release 1.0 (Jul 2013), which integrates 18 disease associated databases, consists of 4,465,131 entries dealing with 139,365 DR-MPEs and 3,824 human diseases.

## Materials and Methods

### Disease-related databases

18 disease-related open source databases (Table 1) are integrated in our database. The DR-MPEs in these databases are divided into 10 categories: gene, protein, enzyme, genetic variation, microRNA, metabolite, drug, phenotype, virus, and environment. The relations between DR-MPEs and diseases have been described in the 18 databases. We extract the DR-MPEs and remove the redundant information among these databases. For example, the relations between genes and diseases are from OMIM, GeneRIF [23], GAD [24], CTD and SpliceDisease [25]. The relations between genetic variations and diseases are from NHGRI GWAS Catalog, Cancer GAMAdb [26], GWASdb [27], DistiLD [28] and dbCRID [13].

**Table 1:** Disease-related databases integrated into SIDD.

Database Name	Database Category	URL	Disease Vocabulary	Number of Diseases
GeneRIF	Gene and Disease	http://www.ncbi.nlm.nih.gov/gene/about-generif	DO	1966
OMIM	Gene and Disease	http://www.omim.org/	OMIM	1819
GAD	Gene and Disease	http://geneticassociationdb.nih.gov/	MeSH	2194
SpliceDisease	Gene and Disease	http://202.38.126.151:8080/SDisease/	MeSH	547
CTD	Gene and Disease	http://ctdbase.org/	MeSH	2227
dbCRID	Genetic variation and Disease	http://dbcrid.biolead.org/index.php	DO	513
Cancer GAMAdb	Genetic variation and Disease	http://www.hugenavigator.net/CancerGEMKB/home.do	DVSDs	106
GWASdb	Genetic variation and Disease	http://jjwanglab.org:8080/gwasdb/	DVSDs	521
DistiLD	Genetic variation and Disease	http://distild.jensenlab.org/	DVSDs	338
NHGRI GWAS Catalog	Genetic variation and Disease	http://www.genome.gov/gwastudies/	DVSDs	393
miR2Disease	MicroRNA and Disease	http://www.mir2disease.org/	DO	260
HMDD	MicroRNA and Disease	http://202.38.126.151/hmdd/mirna/md/	MeSH	592
UniProtKB	Protein and Disease	http://www.uniprot.org/	MeSH	1275
HMDB	Metabolite and Disease	http://www.hmdb.ca/	OMIM	427
BRENDA	Enzyme and Disease	http://www.brenda-enzymes.org/	DVSDs	2956
DR.VIS	Virus and Disease	http://www.scbit.org/dbmi/drvis	DVSDs	35
GAD	Environment and Disease	http://geneticassociationdb.nih.gov/	MeSH	710
CTD	Environment and Disease	http://ctdbase.org/	MeSH	2392
HPO	Phenotype and Disease	http://www.human-phenotype-ontology.org/	OMIM	1901
PharmGKB	Drug and Disease	http://www.pharmgkb.org/index.jsp	MeSH	337

This table provides the name, category, vocabulary and URL of disease-related databases integrated into SIDD. All the databases are divided into 10 major categories. In addition, the last column presents the number of diseases documented in each database.

The disease terms in the different databases are from different disease vocabularies: MeSH, OMIM, DO and DVSDs (Disease Vocabularies for Specific Databases). DO is used to organize the disease terminologies in miR2Disease and dbCRID; MeSH is used in GAD, CTD, HMDD, SpliceDisease and PharmGKB [29]; OMIM, HMDB and HPO use terminologies included in OMIM; and the other 8 databases adopt other disease vocabularies or define disease terms in their own ways. It’s a key to unify the disease terms in different databases to DO. In previous researches, disease terms in GeneRIF and UniProtKB [30] have been annotated to DO and MeSH, respectively [31], [32]. For disease terms annotated in the 2 databases, we incorporate their results directly into SIDD.

### Integration of disease-related databases

The first challenge to integrate several disease-associated databases is to unify disease vocabularies from different databases. Here, we take the following mapping strategy to solve the problem. Firstly, the disease terms from 18 databases are mapped to DO by program as direct mapping, if the disease term is the cross reference [14] or synonymous of DO term. In particular, synonymous of disease term pairs are manually checked. The remaining disease terms in MeSH and OMIM are defined as indirect mapping if their ancestors can be mapped to DO terms. The other indirect mapping includes disease terms from DVSDs which are not mapped to DO. They are built into DO through partial matching and manual check. This partial matching process is implemented through Open Biomedical Annotator (OBA) [33], which is an ontology-based web service that annotates public datasets with biomedical ontology concepts based on their textual metadata. Most of the disease terms in different vocabularies are unified to DO after mapping. Under the mapping strategy, disease terms with direct mapping relationship are totally equivalent. In contrast, indirect mapping reflects inclusion relationship. Disease terms in vocabularies are amplified by indirect mapping to DO.

MeSH is a controlled thesaurus of over 26,000 primary terms and divided into 16 sections. We select two disease-related sections: Diseases [C] and Mental Disorders [F03], which include 4,668 disease terms. The process of mapping from MeSH to DO has three major steps: 1) mapping from cross reference (MFR); 2) mapping from synonyms (MFS); 3) mapping from inferring (MFI). The first two steps are direct mapping and the last step is indirect mapping. In the MFR step, 2,701 MeSH disease terms (Table 2) are mapped to DO using their existing cross-reference in the DO database. For those unmapped disease terms in the MFR step, we search for their synonyms for any documented entry in the DO and MeSH databases by exact matching. Then further map those concepts by synonym relationship in the MFS step. The mapping results are manually checked to avoid the mapping errors as much as possible. Consequently, 236 diseases (Table 2) with synonymous concepts are kept. To the remaining disease terms in MeSH, their nodes in the MeSH ontology tree are mapped from the closest ancestor nodes, which can directly be mapped to DO. ‘is_a’ (i.e. ‘is a subclass of’) characterizes a core relation in MeSH and DO. It refers to a subset or an inclusion relation [34]. For instance, in MeSH, ‘Lupus Erythematosus, Discoid (D008179)’ is a ‘Lupus Erythematosus, Cutaneous (D008178)’ which is synonymous of ‘DOID:0050169’ in DO.

**Table 2:** The number of disease terms mapped to DO.

Disease Vocabulary	MFR	MFS	MFI	RCA
MeSH	2701	236	1347	0
OMIM	1668	378	2140	0
DVSDs	0	654	0	1685
Total	4501	1268	3487	1685

The disease terms of MeSH, OMIM and DVSDs are mapped to DO by direct mapping (MFR and MFS) and indirect mapping (MFI and RCA). The number of mapped diseases in each step is presented.

Similar mapping process from MeSH to DO, 2,046 OMIM disease terms (Table 2) can be directly mapped to DO through cross-references and synonyms. OMIM documents the disease concepts, but it does not provide connections between similar diseases. We use OMIM-MeSH combined vocabulary [18], which is a manually created, practical, structured vocabulary, by curating the association of MeSH with OMIM to indirectly map disease concepts. 2,140 disease terms (Table 2) match to the semantically closest ancestor, which can directly map to DO. In total, 4,186 OMIM disease terms can map to DO vocabulary.

For six disease-related databases using DVSDs, we use OBA [33] to annotate a disease term with DO, if it is not included in the DO database. We manually check all the OBA annotation results for removing error annotations. In cases where multiple DO terms along a branch of DO tree mapped by a disease term, only the most specific mapped DO term are kept. This mapping is named as Mapping with Reviewed Computational Annotation (RCA), and is indirect form of mapping. Thus far, four types of mapping (MFR, MFS, MFI and RCA) are formed for associating disease terms with DO. The software of mapping program is implemented by JAVA 6.0, which is freely available at http://mlg.hit.edu.cn/SIDD/rsfdb.jsp

In addition to unify disease terms, another issue also needs to be addressed for integrating these databases, which is how to filter out redundant records. Many records in different databases are from the same reference or describe the same relationship between DR-MPE and disease. For example, the same relationship between breast cancer and gene *AKT1* is documented in GeneRIF, OMIM, CTD and GAD. These records are stored in an unified format then merged into one record. Each record in this format includes seven items: disease name, disease ID, DR-MPE ID, DR-MPE symbol, DR-MPE type, mapping type, and source. Among them, the first two items mean disease name and identifier described in DO. The third, fourth and fifth items represent the identifier, symbol and types of DR-MPEs, respectively. The sixth item gives the type of mapping (MFR, MFS, MFI and RCA) from disease term to DO. The seventh item is the name of databases. There are various IDs and symbols extracted from the 18 databases for describing DR-MPE ID and DR-MPE symbol. For example, NCBI Entrez Gene ID and HGNC Gene Symbol are extracted from GeneRIF, OMIM, GAD (Gene), CTD (Gene) and SpliceDisease; dbSNP ID is extracted from Cancer GAMAdb, GWASdb, DistiLD, NHGRI GWAS Catalog; miRBases's microRNA ID and symbol are extracted from HMDD and miR2Disease. The redundant records describing the same DR-MPE and disease are easily removed after all the original records are represented as unified format. All non-redundant records are stored in an index file.

### Process of mapping validation

Mapping process includes four steps: MFR, MFS, MFI, RCA. MFR step uses DO's cross mapping results [14]. In MFI step, disease terms in ontology tree are mapped from the closest ancestor nodes, which can directly be mapped to DO [34]. Therefore, the mapping result in MFR and MFI steps are not manually checked.

The MFS and RCA mapping results are manually validated (Figure S1). The MFS mapping result of disease term pairs and the RCA mapping result of disease term pairs are equally divided into four parts. Each part is independently checked by two medical Ph.D. students from Cancer Hospital of Harbin Medical University. Totally four students participate this manual checking process.

For the MFS mapping result, the disease term pair is scored with ‘1’ if the two different terms are considered as the same disease in manual check, and ‘0’ otherwise. For the RCA mapping result, the disease term pair is scored with ‘1’ if disease term from DVSDs (Disease Vocabularies for Specific Databases) was included by DO, and ‘0’ otherwise. In both MFS and RCA result manual checking, only mapping terms scored with double “1” from both students are kept in the database. This stringent criteria warrant a high accuracy for our mapping process.

### System design and implementation

Three major steps for integrating the relationship between diseases and DR-MPEs in SIDD are as follows. (1) extracting the DR-MPE records from 18 source database, (2) mapping all disease terms to DO, (3) filtering out the redundant records among the same DR-MPE type databases. MySQL version 5.5.1 has been employed to manage all results of the three steps. The whole SIDD framework is running on our web server (8-core (2.0 GHz) processors with 64 Gigabytes of RAM).

## Results

### Mapping validation

The MFS mapping result of 1,362 disease term pairs and the RCA mapping result of 2,340 disease term pairs are manually checked. The MFS mapping result of 1,268 (93.1%—1,268/1,362) disease term pairs and the RCA mapping result of 2,302 (98.3%—2,302/2,340) disease term pairs scored with double ‘1’ are remained in SIDD (Table 3), the left of them are removed. More detailed checked mapping results are provided in supporting information (Dataset S1).

**Table 3:** The manual checking result of MFS and RCA mapping.

Mapping type	Total	Scored with double ‘1’	Scored with ‘1’ and ‘0’	Scored with double ‘0’
MFS	1362	1268	58	36
RCA	2340	2302	33	5

### Database content

**Figure 1:**
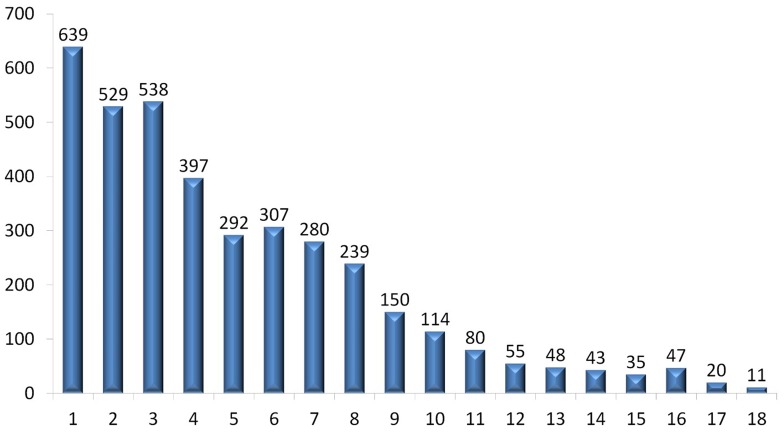
Distribution of diseases integrated in SIDD. The height of each bar (vertical axis) represents the amount of unique diseases appears in 1, …,18 databases, for example, the first bar means one of the 639 diseases only appears in one of the databases, rather than in the other 17 databases. The second bar means one of the 529 diseases only appears in two of the databases.

In the current release version (Jul 2013) of SIDD, 18 disease-related databases are integrated, and 4,465,131 relationships between 3,824 diseases and 139,365 DR-MPEs are extracted and inferred from the original databases. In detail, 1,036,994 relationships between diseases and DR-MPEs are extracted, and 3,428,137 relationships are inferred by ‘is_a’ relationship of DO. Detailed statistics of SIDD database are given in the Table S1. Among 3,824 diseases, only 639 diseases (16.7%) are presented in only one database (Figure 1), and all the other 3,185 diseases (83.3%) are covered in at least two databases. In particular, 1,429 diseases (37.4%) are included in more than 5 databases. For instance, some well-studied diseases - prostate cancer, breast cancer, diabetes mellitus, and heart disease - are documented in more than 16 databases.

### Web interface

**Figure 2:**
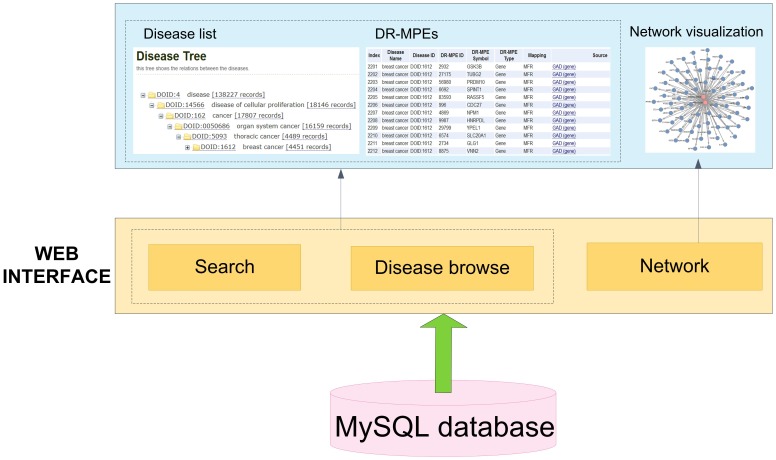
System overview of SIDD database. A system overview of the SIDD database displays the data sources and web interface features.

A web interface has been designed for accessing database, which is constructed by JSP and Servlet. The web interface offers three main functionalities (shown in the top of Figure 2). First, Disease term can be searched and shown in the DO tree. Second, DR-MPEs can be browsed and downloaded. Third, network containing diseases and their co-related DR-MPEs can be visualized in the webpage. In addition, querying and submitting the mappings from disease terms to DO are also supported.

### Disease browse page

**Figure 3:**
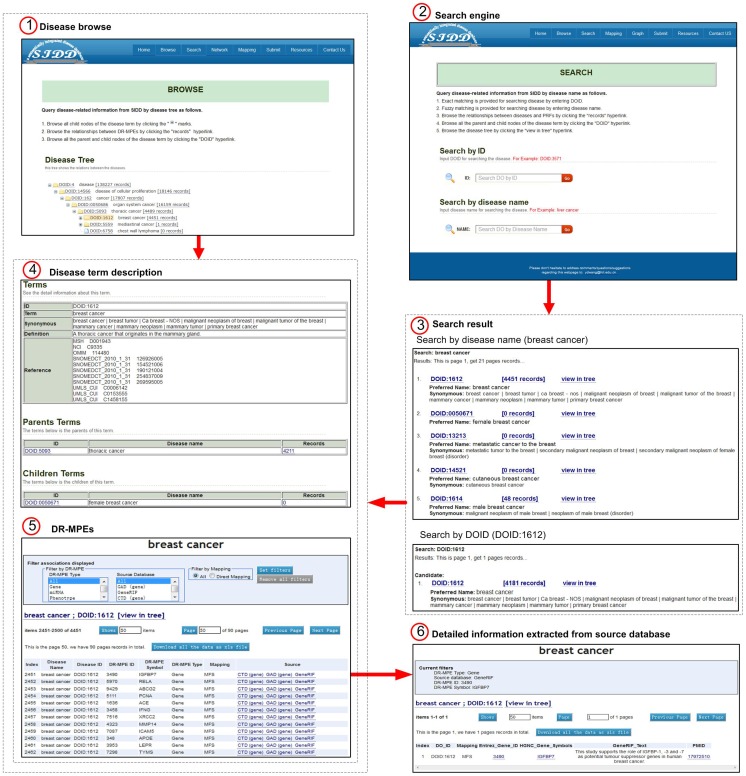
Schematic workflow of SIDD's searching and browsing. (1) Disease browse page. Disease terms are expanded in the DO tree, each of which includes 2 hyperlinks, DOID and the records of disease related factors. (2) Search engine page. Disease terminologies can be accessed by submitting DOID or disease name. (3) Search result page. Corresponding disease terms are listed after search, each of which includes 3 hyperlinks, such as DOID, the records of disease related factors, and view in tree. Disease terms will be shown in the DO tree by clicking the hyperlink in ‘view in tree’. (4) disease term description page. All parent nodes and child nodes of the disease term that are accessed from ‘Disease browse’ or ‘Search engine’ page are listed by clicking the hyperlink in DOID; Corresponding DR-MPEs information about the number of the disease related factors in specific databases are listed by clicking the hyperlink in the records of disease related factors from ‘Disease browse’ or ‘Search engine’ page. (5) DR-MPEs page and (6) Detailed information extracted from source database. More specific association records between disease and DR-MPEs extracted from source databases are accessible when clicking on the links in the Source column.

SIDD provides a disease ontology browse page that shows the hierarchical structure among the disease terms (Figure 3). For each disease shown in the page, a hyperlink is created for accessing detailed information of this disease term in the DO database, and the number of DR-MPEs record that links to a global view of diseases and DR-MPEs associations in SIDD is presented behind the disease term.

### Search page

SIDD provides a search engine that allows the user to query the database through the DOID and disease name, which adopt exact and fuzzy matching to disease vocabulary respectively. Once a certain disease name is received as a query term, SIDD will return disease terminologies that are the most similar. The matching disease terminologies will be listed in the webpage, and hyperlinked to the disease information, disease ontology tree (disease browse page) and a global view of relationships between diseases and DR-MPEs in SIDD. In the DR-MPEs page, user can access all associated DR-MPEs of disease, including DR-MPE names, mapping types, source databases, and so on. Furthermore, user can view the detailed DR-MPE information extracted from the source databases through the hyperlink at the source column. DR-MPEs' ID, PubMed ID, accession number of relationship included in the detailed record can link to the original database. To query and browse DR-MPE easily, an advanced search which can filter the query results by DR-MPE type, source database and mapping type is provided. In addition, user can download the relationships data from the website.

### Network visualization page

**Figure 4:**
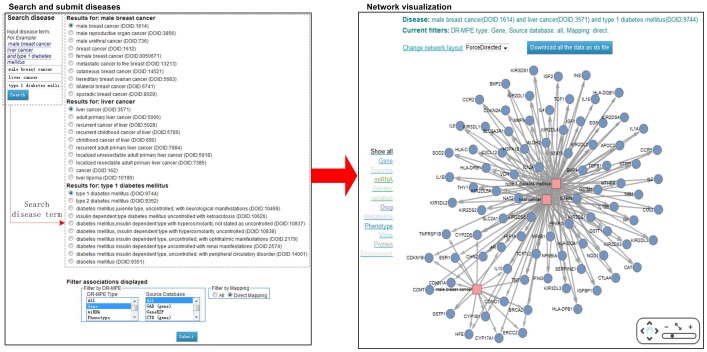
Schematic workflow of SIDD's network visualization. (1) Two or three interested disease terms are accessed by inputting their disease names. (2) select the disease terms from the corresponding disease terms list. (3) a network about these diseases and their co-related DR-MPEs are visualized.

Network shows the connections among diseases by their co-related DR-MPEs, which is visualized in the visualization webpage by Cytoscape Web plugin [35]. There are two steps for generating the network (Figure 4): (1) Two or three disease names are input for querying, and ten most relevant disease names retrieved from DO are listed for each of them. (2) User can select one disease from each disease list, and filter the SIDD database by DR-MPE type, source database or mapping type for viewing the DR-MPEs associations among diseases. After that, a network about these diseases and their co-related DR-MPEs are generated. Each node represents a disease or a DR-MPE, and each edge represents a relationship between a disease and a DR-MPE. The user is also provided with the option to download the association.

### Mapping page

On the mapping page, SIDD provides the function of querying mapping results from MeSH and OMIM to DO. User can access the mapping results by querying DOID or disease name.

### Submit page

SIDD provides a submit page that allows user to submit new mapping from MeSH or OMIM to DO. Once approved by a review procedure, the submitted mapping will be included in the database and made available to the public in the next release.

## Discussion

In SIDD, 68.6% and 48.9% disease terms in MeSH and OMIM can be respectively directly mapped to DO. Therefore, indirect mapping is an effective alternative for establishing the relationship between DO terms and other source databases[14]. For example, ‘Hypobetalipoproteinemia, Familial, Apolipoprotein B (D052476)’ in MeSH is mapped to ‘hypobetalipoproteinemia (DOID:1390)’ in DO by MFI (Figure S2). It means that D052476 is a subset of DOID:1390, and the instances of the former are also instances of the latter [34]. In addition, relationships inferred by ‘is_a’ relationship in DO between DR-MPEs and diseases enrich the content in SIDD. For example, 151 DR-MPEs related to the ‘genetic disease (DOID:630)’ before we used inferring, with 5,474 DR-MPEs being subsequently inferred.

**Figure 5:**
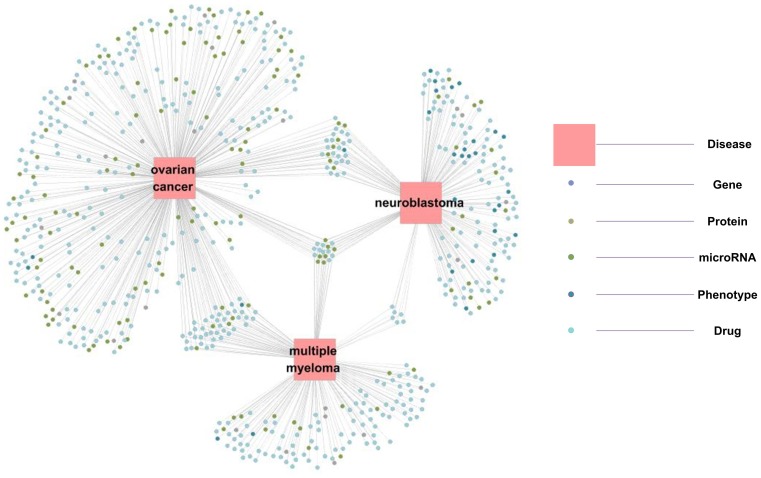
Interaction network demonstrating the relationships between 5 types of DR-MPEs (gene, protein, microRNA, phenotype and drug) and 3 diseases (ovarian cancer, neuroblastoma and multiple myeloma). The large circle corresponds to disease, and the smaller circle in a different color corresponds to different DR-MPEs. An edge indicates a relationship between DR-MPE and disease.

Information in SIDD can describe the relationships between multiple diseases at multiple biological levels. To this end, we create an illustration network in Cytoscape [36] that describes the relationships between 5 types of DR-MPEs (gene, protein, microRNA, phenotype, drug) and 3 well-studied diseases (ovarian cancer, neuroblastoma and multiple myeloma), presenting an intuitive perspective (Figure 5). The circles at the center of the figure highlight 10 genes (*CXCL12*, *MMP2*, *BCL2*, *MYC*, *BIRC5*, *CCND1*, *CDKN2A*, *IGF1*, *SKP2*, *KIT*) and 7 microRNAs (*hsa-mir-17*, *hsa-mir-18a*, *hsa-mir-19a*, *hsa-mir-19b-1*, *hsa-mir-20a*, *hsa-mir-335*, *hsa-mir-92a-1*) related to all 3 diseases. The web site of SIDD currently contains the customized option for generating this type of network. The user also can search for the interested diseases, download the relationships between diseases and DR-MPEs, and create the network using other tools.

As shown in the Figure 2, 3,184 of 3,824 diseases (83.3%) are presented in at least 2 databases. Therefore, SIDD enables researchers to understand disease at multiple biological levels. Furthermore, different types of DR-MPEs could be associated by their co-related disease. We have found evidence from literature for some associations. Two of them are listed as follows. One example is that microRNA *hsa-miR-27a* and gene *PHB, SPRY2* are related with the same disease ‘hepatocellular carcinoma (DOID:684)’ by SIDD. These genes are documented as the target of *hsa-miR-27a-3p* in recent studies [37], [38]. Another example is that drug bevacizumab and gene *VEGF* are co-related with disease ‘ovarian cancer (DOID:2394)’ in SIDD. The gene has been validated as the therapeutic target of bevacizumab [39].

In summary, SIDD is a comprehensive resource that integrates disease-related databases. We believe that it will be of particular value to the life scientists and allows biologists to understand disease at multiple biological levels.

### Availability and Future Directions

The SIDD is available via a web-based interface at http://mlg.hit.edu.cn/SIDD. The database will be updated quarterly to provide information about the relationships between DR-MPEs and diseases. We plan to integrate more disease-related databases into SIDD in the future. We also plan to improve our mapping results of disease vocabularies through manual checking by more biologists.

## Supporting Information

Figure S1 **The process of manual checking.**
(DOCX)

Figure S2 **An example of MFI from MeSH to DO.**
(DOCX)

Table S1 **Statistics of SIDD database.**
(DOCX)

Dataset S1 **The detailed manual checking result of MFS and RCA mapping.**
(XLSX)
