# Supplementary material for: SIDD: A Semantically Integrated Database towards a Global View of Human Disease
Source: PLoS One. 2013 Oct 11;8(10):e75504. doi: 10.1371/journal.pone.0075504 (PMC3795748; doi:10.1371/journal.pone.0075504)
Supplement: Figure S1 — The process of manual checking. (DOCX) [file pone.0075504.s001.docx]

**Figure S1 (Supplementary Figure 1). The process of manual checking.**

The MFS mapping result of 1,362 disease term pairs and the RCA mapping result of 2,340 disease term pairs are equally divided into four parts. Each part is independently checked twice in two stages. In the first stage, each student checks one part. In the second stage, each student checks one different part of them.
