# Supplementary material for: SIDD: A Semantically Integrated Database towards a Global View of Human Disease
Source: PLoS One. 2013 Oct 11;8(10):e75504. doi: 10.1371/journal.pone.0075504 (PMC3795748; doi:10.1371/journal.pone.0075504)
Supplement: Figure S2 — An example of MFI from MeSH to DO. (DOCX) [file pone.0075504.s002.docx]

**Figure S2 (Supplementary Figure 2). An example of MFI from MeSH to DO.**

The mapping disease term pairs are as follows:

MFR: Hypolipoproteinemias (D007009) to DOID:1387

MFR: Hypobetalipoproteinemias (D006995 ) to DOID:1390

MFI: Hypobetalipoproteinemia, Familial, Apolipoprotein B (D052476) to DOID:1390.
