# Supplementary material for: SIDD: A Semantically Integrated Database towards a Global View of Human Disease
Source: PLoS One. 2013 Oct 11;8(10):e75504. doi: 10.1371/journal.pone.0075504 (PMC3795748; doi:10.1371/journal.pone.0075504)
Supplement: Table S1 — Statistics of SIDD database. (DOCX) [file pone.0075504.s003.docx]

**Table S1 (Supplementary Table 1). Statistics of SIDD database.**

The third and fourth columns represent the number of records documented in the each original database, and that have been extracted by SIDD database, respectively. The fifth column corresponds to the relationships between DR-MPEs and diseases extracted from the original databases. For some databases, such as OMIM, GAD, the number of relationships are greater than the number of records because certain records in the original database contain more than one disease. The sixth column records the number of all extracted and inferred relationships in SIDD. The seventh column is the number of DR-MPEs that integrated in SIDD.

| Database name | DR-MPEs type | Number of records in original database | Number of records in SIDD | Number of relationships between DR-MPEs and diseases in original database | Number of relationships between DR-MPEs and diseases in SIDD | Number of DR-MPEs |
| --- | --- | --- | --- | --- | --- | --- |
| GeneRIF | Gene | 52292 | 49924 | 49924 | 265046 | 5314 |
| OMIM | Gene | 7293 | 5693 | 7993 | 40752 | 3918 |
| GAD | Gene | 127596 | 115102 | 225305 | 940636 | 11295 |
| SpliceDisease | Gene | 2375 | 2264 | 2750 | 14159 | 318 |
| CTD | Gene | 19457 | 18557 | 24397 | 120930 | 6517 |
| dbCRID | Genetic Variation | 3123 | 2366 | 2384 | 17985 | 2365 |
| Cancer GAMAdb | Genetic variation | 9893 | 9761 | 9779 | 56723 | 1211 |
| GWASdb | Genetic variation | 165189 | 61781 | 66795 | 402032 | 47809 |
| DistiLD | Genetic variation | 78840 | 76647 | 315314 | 1036467 | 70912 |
| NHGRI GWAS Catalog | Genetic variation | 8205 | 3791 | 4005 | 25083 | 3108 |
| miR2Disease | MicroRNA | 2183 | 2183 | 2183 | 15969 | 406 |
| HMDD | MicroRNA | 7111 | 6956 | 9792 | 50133 | 516 |
| UniprotKB | Protein | 2934 | 2919 | 3881 | 21564 | 1955 |
| HMDB | Metabolite | 1268 | 1099 | 1216 | 7268 | 500 |
| Brenda | Enzyme | 106287 | 103068 | 131411 | 645049 | 1269 |
| DR.VIS | Virus | 566 | 566 | 632 | 3453 | 11 |
| GAD | Environment | 4496 | 3843 | 8075 | 32210 | 714 |
| CTD | Environment | 75302 | 68160 | 81379 | 383122 | 7370 |
| HPO | Phenotype | 55087 | 46257 | 86180 | 368220 | 5803 |
| PharmGKB | Drug | 4375 | 3132 | 3599 | 18330 | 283 |
| Total | All | 733872 | 584069 | 1036994 | 4465131 | 139365 |
